# The impact of the introduction of ten- or thirteen-valent pneumococcal conjugate vaccines on antimicrobial-resistant pneumococcal disease and carriage: A systematic literature review

**DOI:** 10.7189/jogh.13.05001

**Published:** 2023-02-17

**Authors:** Rita Reyburn, Jaclyn Maher, Claire von Mollendorf, Amanda Gwee, Kim Mulholland, Fiona Russell, ARI Review group, Trevor Duke, Hamish Graham, Steve Graham, Amy Gray, Maeve Hume-Nixon, Saniya Kazi, Priya Kevat, Eleanor Neal, Cattram Nguyen, Alicia Quach, Kathleen Ryan, Patrick Walker, Chris Wilkes, Poh Chua, Yasir Bin Nisar, Jonathon Simon, Wilson Were

**Affiliations:** 1 Murdoch Children’s Research Institute, Melbourne, Victoria, Australia; 2 Faculty of Medicine, Dentistry and Health Sciences, The University of Melbourne, Melbourne, Victoria, Australia; 3 Department of Paediatrics, The University of Melbourne, Melbourne, Victoria, Australia; 4 The Royal Children's Hospital, Melbourne, Victoria, Australia; 5 London School of Hygiene and Tropical Medicine, London, UK

## Abstract

**Background:**

A systematic review in 2019 found reductions in antimicrobial resistance (AMR) of pneumococcal vaccine serotypes following pneumococcal conjugate vaccine (PCV) introduction. However, few low- or middle-income countries were included as not many had introduced higher valent PCVs (PCV10 or PCV13). The aim of our review is to describe AMR rates in these samples following the introduction of PCV10 or PCV13.

**Methods:**

We conducted a systematic literature review of published papers that compared AMR for invasive pneumococcal disease (IPD), otitis media (OM) and nasopharyngeal carriage (NPC) samples following introduction of PCV10 or PCV13 to the pre-PCV period. Included studies published from July 2017 to August 2020 had a post-licensure observational study design and reported on our defined outcomes: IPD, OM, NPC and other (sputum or mixed invasive and non-invasive pneumococcal) isolates from people of all ages. Rates of AMR in the pre- and post-period were extracted.

**Results:**

Data were extracted from 31 studies. Among IPD isolates, penicillin AMR rates following PCV10 or PCV13 introduction declined in 32% (n = 9/29) of included studies, increased in 34% (n = 10/29) and showed no change in 34% (n = 10/29). Cephalosporins AMR declined in 32% (n = 6/19) of studies, increased in 21% (n = 4/19) and showed no change in 47% (n = 9/19). Macrolides AMR declined in 33% (n = 4/12) of studies, increased in 50% (n = 6/12), and showed no change in 17% (n = 2/12). AMR to other antibiotics (including multidrug resistance) declined in 23% (n = 9/39) of studies, increased in 41% (n = 16/39) and showed no change in AMR in 36% (n = 14/39). There were no obvious differences between AMR; in setting which used PCV10 vs PCV13, according to time since PCV introduction or by World Bank income status of the respective country. The only study including OM isolates found no change in penicillin resistance. There were few studies on AMR in NPC (four studies), OM (one study) or other isolates (five studies). The results followed similar patterns to IPD isolates.

**Conclusions:**

We observed considerable heterogeneity in the findings between and within studies, e.g. no evidence of reduction in amoxicillin AMR with an increase in macrolides AMR. Reasons for such diverse findings include the period covered by different studies and variation in other pressures towards AMR.

Antimicrobials have been central to the treatment of infection for more than 80 years but their increasing use has stimulated selection of antimicrobial resistance (AMR) strains among common bacterial pathogens [1]. As new antimicrobial agents are introduced, the degree and diversity of resistance in pathogens has also increased [2].

The cost of AMR to human life is difficult to accurately measure, but it has been estimated that in 2015 in the European Union, at least 700 000 people each year became infected with antimicrobial resistant bacteria, and at least 33 000 died as a direct result of these infections [3].

Streptococcus pneumoniae is among the commonest human infections for which antimicrobials are prescribed and to which resistance continues to rise. However, resistance patterns have been modified during the last two decades by many factors, including the introduction of 7-valent pneumococcal conjugate vaccine (PCV7), which covers the most common pneumococcal serotypes causing invasive disease [4,5]. The use of the first generation PCV7 in children in the United States, containing serotypes 4, 6B, 9V, 14, 18C, 19F and 23F, led to a reduction in pneumococcal disease due to these AMR serotypes, particularly those resistant to penicillin and/or erythromycin [5]. Subsequently, non-PCV7 serotypes with higher levels of AMR emerged (e.g. serotypes 19A and 7F), and this limited the overall benefit of PCV7 in reducing the burden of pneumococcal disease [6]. The next generation higher valency vaccines, 10-valent PCV (PCV10, *Synflorix*) and 13-valent PCV (PCV13, *Prevnar13*®), have now been introduced with the latter containing six new serotypes in addition to those in PCV7 (1, 3, 5, 6A, 7F, and 19A).

A previous systematic review reported on the effect of PCV10 or PCV13 implementation in routine infant immunisation schedules on AMR invasive pneumococcal disease (IPD), otitis media (OM), and nasopharyngeal carriage (NPC) in children and adults using literature published between 2008 and 2017. Results showed that in countries with relatively high prior pneumococcal AMR, PCV13 childhood vaccination programs have reduced AMR IPD, OM, and NPC in children and IPD in adults [7]. The effectiveness of PCV13 against serotype 19A was likely an important contributing factor. This published review used studied published up to one June 2017 and included few studies from low- and middle-income countries (LMICs) using higher valency PCVs as national introduction of PCV10 and PCV13 occurred more recently. A more recent systematic review included data on paediatric isolates from 104 countries, and demonstrated reduction in proportions of pneumococci showing non-susceptibility to penicillin 11.5% (95% confidence interval (CI) = 8.6-14.4), sulfamethoxazole-trimethoprim 9.7% (95% CI = 4.3-15.2), and third-generation cephalosporins 7.5% (95% CI = 3.1-11.9), over the 10 years after implementation of any PCV product [8]. This review did not include data on isolates from adults.

The aim of this systematic literature review is to describe the impact of PCV10 or PCV13 as part of the national immunisation program on AMR rates in IPD, OM, and NPC samples from people of all ages.

## METHODS

This study was conducted following the Preferred Reporting Items for Systematic Reviews and Meta-Analyses (PRISMA) statement. The protocol is available upon request from the authors.

### Literature search

A systematic literature review was performed to identify data from published studies on the impact of PCV10/13 on AMR rates in IPD, OM and NPC samples taken from children and adults compared to a pre-PCV period. The literature used in this review was obtained through electronic searches of MEDLINE (Ovid), Embase (Ovid) and Cochrane Library databases using Medical Subject Headings (MeSH), thesaurus terms and keywords. Details of the search terms and keywords are presented in Table S1 in the **Online Supplementary Document**. PubMed was additionally searched, using keywords only, to source any electronic publications as well as items not indexed in Medline. There was no restriction regarding languages included in the search terms; however, we did not specifically search non-English languages.

### Inclusion and exclusion criteria

We included studies carried out in countries which have introduced PCV10/13 into their routine national immunisation schedule. The review focused on the two commercially available vaccines, PCV10 (Synflex) and PCV13 (Prevenar 13®), and considered any immunisation schedule: two primary doses plus a booster (2 + 1) or three primary doses with or without a booster (3 + 1 or 3 + 0), with or without catch-up. PCV13 or PCV10 uptake in the recommended immunisation population of at least 70% during the reported study period was required for inclusion. Observational studies, including before-after and interrupted time series, were targeted. The outcomes of interest were: AMR rates in IPD, OM and NPC. No restrictions were applied to the antibiotics that were assessed in the studies or the guidelines and criteria that were used to define antibiotic resistance or non-susceptibility. Reference lists of identified reviews were screened for publications meeting the inclusion criteria that had not been found in the electronic search. Studies published between July 2017 and August 2020 were included. The following exclusion criteria were applied: randomised controlled trials were excluded as the aim was to evaluate the impact of PCV13 and PCV10 in national immunisation programs. In addition, case-control studies, cross-sectional studies, case series and case reports as well as studies that only reported data before or after PCV introduction but not for both periods were also excluded. To allow sufficient time for vaccine impact, we excluded studies which assessed AMR rates less than one year post-PCV10/13 introduction, as well as studies which that only compared PCV13 or PCV10 to PCV7 rather than to the pre-PCV period. We excluded studies in which only the number of cases, and not rates, were presented since these do not allow a standard comparison with other studies.

### Study selection

Citations were screened by two independent reviewers in a two-step approach. First, two independent reviewers screened the titles and abstracts for their relevance based on the inclusion/exclusion criteria. For publications which were unclear from the title/abstract whether they met these criteria were included for full-text reading. Second, full-text articles were reviewed independently by two reviewers. Reasons for excluding studies were recorded.

### Data collection and assessment of study quality

Data extraction was done by one reviewer and reviewed by the second reviewer. Forms developed specifically for this systematic review were used to extract data for standardised variables across the studies. Standard extracted data was based on the Strengthening the Reporting Observational Studies in Epidemiology (STROBE) statement, including the study population (age, and number of isolates), study design, study period, setting and location (national or regional), evaluated pneumococcal disease syndrome or site of collection of isolates, whether The Clinical & Laboratory Standards Institute (CLSI) guidelines were used for break points to define AMR or non-susceptibility, and introduction date and schedule of the pneumococcal vaccination program. Outcomes included the proportion/percentage or incidence of antimicrobial-resistant pneumococcal disease (pneumococcal disease includes IPD, defined as isolation of pneumococci from a normally sterile site, or OM or NPC) before and after the implementation of PCV10 or PCV13 childhood immunisation program. All studies were independently assessed for quality considering the items of structured quality scoring systems as checklists. The level of risk of bias in study analysis was assessed for each study using the Effective Public Health Practice Project (EPHPP) Quality Assessment Tool for Quantitative Studies. Internal validity of each study was evaluated considering eight methodological domains: selection bias of study participants, study design, confounders, blinding, data collection methods, withdrawals of study participants, intervention integrity and analysis.

### Data analysis

Study characteristics including design, country, type and schedule of PCV introduced, data source, and endpoints were summarised.

For all studies, the main measure of interest was the AMR rates before and after PCV10 or PCV13 introduction. For time series studies, the outcomes were reported as either the percent reduction in rates when modelling observed rates against predicted rates of disease or resulting from a percent change in incidence rates when comparing the post and pre-vaccination periods. In before-after studies, vaccine effects were reported as percent change in rates (prevalence, incidence, or mortality rate reduction). For studies reporting only percentage of AMR isolates in the pre-PCV and the post-PCV10 or PCV13 period, this data was presented without a rate reduction. For studies which reported data for multiple years, only data for the earliest year in the pre-PCV period and data for the latest year for the post-PCV13 or PCV10 were presented to allow maximum time for the vaccine to have an impact in the population.

The outcome of interest was AMR for any antibiotic. Studies were then categorised by the following antibiotic classes: penicillin, cephalosporin, macrolide and other. Multidrug resistance was defined as AMR to three or more antibiotic classes. Where possible, the use of standard laboratory methods, including break points was assessed by whether studies used the CLSI Guidelines [9]. Data on the defined daily doses (DDD) of antibiotics consumed per 1000 inhabitants per day for any antibiotic for countries were extracted from the World Health Organization (WHO) report on surveillance of antibiotic consumption 2019 and included in the analysis, if available [10], as this is a proxy for local antibiotic use and analysis was done to determine if DDD was coincided with a change in AMR.

## RESULTS

### Literature search

**Figure 1:**
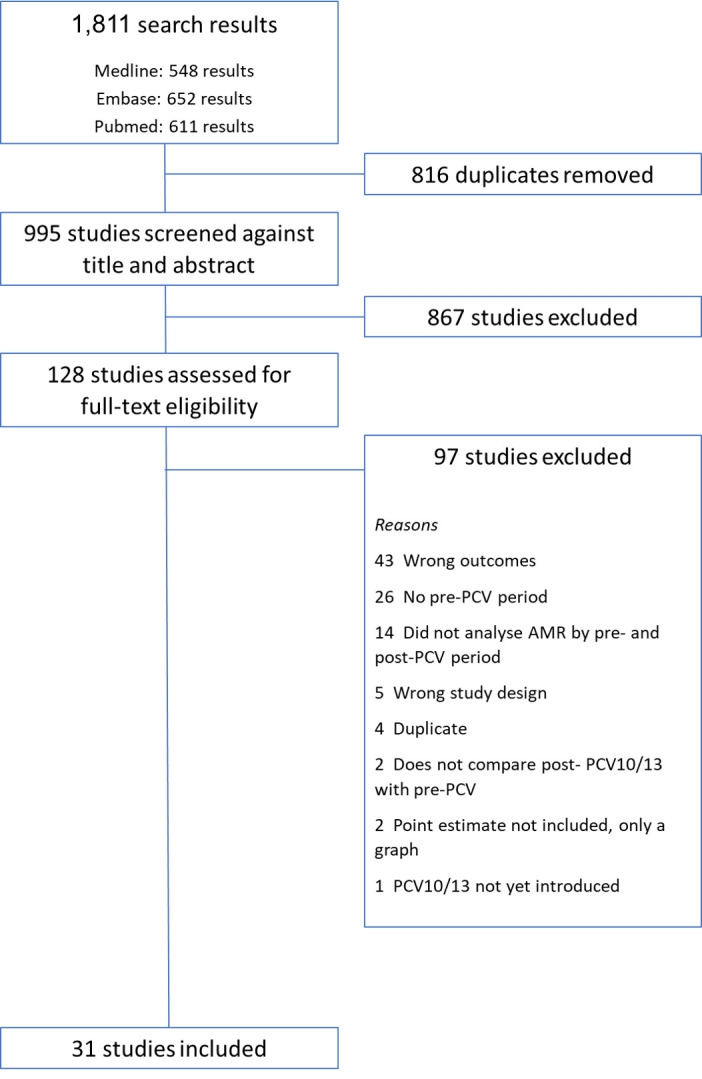
Flow diagram of the study selection process.

The literature search identified a total of 1811 articles. After excluding 816 duplicates, a total of 995 papers were screened by title and abstract and 128 studies were subsequently considered in the full-text review. A total of 31 studies met the inclusion criteria and were included in the final review. For a flow diagram of the study selection process see **Figure 1**.

### Study characteristics

The characteristics of the 31 studies included are summarised in Tables S2-S5 in the **Online Supplementary Document** [11-41]. Of the 31 studies included, 21 studies (68%) assessed changes in AMR rates among IPD isolates, five (16%) among NPC isolates, three (10%) among non-invasive and IPD isolates combined, one (3%) among OM isolates and one (3%) among sputum and specimens of transtracheal aspiration or bronchoscopy from adults with respiratory tract infections.

One study was from a low-income country (LIC), two (6%) studies were from a lower-middle income country (LMIC), 13 (42%) studies were from upper-middle income countries (UMIC), 14 (45%) studies were from a high-middle income country (HMIC) and one included data from countries from multiple World Bank income status. Twenty-two (71%) studies included countries that had introduced PCV13, seven (23%) studies included countries which had introduced PCV10, one (3%) setting, Greece, introduced both PCV10 and PCV13 and one (3%) study was from a number of countries which introduced either PCV10 or PCV13. Twenty-nine (93%) studies were rated as moderate risk of bias, due to the study design, observational cohort, and lack of assessment of confounders, while two (7%) were assessed to be strong/moderate risk of bias.

Thirty studies estimated AMR rates in the pre- and the post- PCV10/13 periods. Of these, 18 (58%) used a significance test to assess if the difference observed was due to chance. Only one study included an estimate in the percent reduction from the pre-PCV period to the post-PCV10/13 period, which calculated a rate difference of 31% and a 5% increase in penicillin and cefotaxime resistance, respectively among IPD isolates [4]. As such, studies could only be summarised according to whether they observed reductions, increases or no change in AMR in the period following the introduction of PCV10 or PCV13. Among the 30 studies, 25 (83%) reported using the CLSI guidelines to determine AMR.

### Changes in AMR rates for IPD isolates

There were 29 results from 22 studies on the change in penicillin resistance or non-susceptibility. Among the 29 results, nine (32%) studies observed reductions in AMR rates in the post-PCV10/13 period, 10 (34%) observed increases and 10 (34%) observed no change (**Table 1**).

**Table 1:** Results of studies displaying the rates of resistant or non susceptible invasive pneumococcal disease isolates in the pre- and post-PCV10/13 periods

ID	Author	Country	Penicillin family (penicillin, amoxicillin, amoxicillin-clavulanate (augmentin), ampicillin, dicloxacillin, nafcillin, and piperacillin-tazobactam (zosyn))					Cephalosporin family (all antibiotics starting with Cef)							Macrolide family (erythromycin, roxithromycin, azithromycin and clarithromycin)					Other				
			**Pre-PCV antibiotic resistance**	**Post-PCV antibiotic resistance**	***P*-value for comparison of pre- vs post- antibiotic resistance**	**Increase, decrease either *P* < 0.05 or + / - 5%**	**Pre-PCV antibiotic resistance**		**Post-PCV antibiotic resistance**	**Percent change in antibiotic resistance**		**Increase, decrease either *P* < 0.05 or + / - 5%**		**Pre-PCV antibiotic resistance**		**Post-PCV antibiotic resistance**	**Percent change in antibiotic resistance**		**Increase, decrease either *P* < 0.05 or + / - 5%**	**Pre-PCV antibiotic resistance**	**Post-PCV antibiotic resistance**	**Percent change in antibiotic resistance**	**Increase, decrease either *P* < 0.05 or + / - 5%**
7	Darboe, 2019 [17]	The Gambia	Penicillin R: 0.0% (0/199)	Penicillin R (2014): 3.2% (2/63)		No change														Gentamicin R: 0% (0/199)	Gentamicin R: 0% (0/63)		No change
			Ampicillin R: 0.0% (0/199)	Ampicillin R: 0.0% (0/63)		No change																	
3	Berezin, 2019 [13]	Brazil	Penicillin R (without meningitis):9.1% (n = 18)	Penicillin R (without meningitis): 4.8% (n = 3)	*P* < 0.001	Decrease		Third-generation cephalosporin R: 9.1% (n = 18)	Third-generation cephalosporin R: 1.6% (n = 1)		*P* < 0.052		Decrease										
			Penicillin (with meningitis) 20.2% (n = 40)	Penicillin (with meningitis) 8.0% (n = 5)	*P* = 0.033	Decrease																	
5	Cassiolato, 2018 [15]	Brazil	<5 y Penicillin R: 11.4% (n = 5)	<5 y Penicillin R: 71.4% (n = 65)	<5 y *P* < 0.001	Increase									<5 y Erythromycin R: 20.5% (n = 9)	<5 y Erythromycin R: 85.7% (n = 78)	<5 y *P* < 0.001	Increase		<5 y Trimethoprim-sulfamethoxazole R:65.9% (n = 29)	<5 y Trimethoprim-sulfamethoxazole R: 86.8% (n = 79)	<5 y *P* = 0.020	Increase
			5-49 y Penicillin R: 2.3% (n = 1)	5-49 y Penicillin R: 57.5% (n = 50)	5-49 y *P* < 0.001	Increase									5-49 y Erythromycin R: 16.3% (n = 7)	5-49 y Erythromycin R: 70.1% (n = 61)	5-49 y *P* < 0.001	Increase		5-49 y Trimethoprim-sulfamethoxazole R: 62.8% (n = 27)	5-49 y Trimethoprim-sulfamethoxazole R: 74.7% (n = 65)	5-49 y *P* = 0.434	No change
			≥50 y Penicillin R: 5.9% (n = 1)	≥50 y Penicillin R: 58.9% (n = 43)	≥50 y *P* < 0.001	Increase									≥50 y Erythromycin R: 23.5% (n = 4)	≥50 y Erythromycin R: 76.7% (n = 56)	≥50 y *P* < 0.001	Increase		≥50 y Trimethoprim-sulfamethoxazole R: 52.9% (n = 9)	≥50 y Trimethoprim-sulfamethoxazole R: 78.1% (n = 57)	≥50 y *P* = 0.531	No change
			All ages Penicillin R: 6.7% (n = 7)	All ages Penicillin R: 62.9% (n = 158)	All ages *P* < 0.001	Increase									All ages Erythromycin R: 19.2% (n = 20)	All ages Erythromycin R: 77.7% (n = 195)	All ages Erythromycin *P* < 0.001	Increase		All ages Trimethoprim-sulfamethoxazole R: 62.5% (n = 65)	All ages Trimethoprim-sulfamethoxazole R: 80.1% (n = 201)	All ages *P* = 0.046	Increase
																				<5 y Multidrug-resistant R: 18.2% (n = 8)	<5 y Multidrug-resistant R: 79.1% (n = 72)	<5 y *P* < 0.001	No change
																				5-49 y Multidrug-resistant R: 9.3% (n = 4)	5-49 y Multidrug-resistant R: 63.2% (n = 55)	5-49 y *P* < 0.001	Increase
																				≥50 y Multidrug-resistant R: 23.5% (n = 4)	≥50 y Multidrug-resistant R: 65.8% (n = 48)	≥50 y *P* = 0.002	Increase
																				All ages Multidrug-resistant R: 15.4% (n = 16)	All ages Multidrug-resistant R: 69.7% (n = 175)	All ages *P* < 0.001	Increase
6	Cho, 2017 [16]	Taiwan, China	2010 Penicillin NS (non-meningitis):57.7%	2015 Penicillin NS (non-meningitis): 43.5%		Increase		2010 Cefotaxime NS (non-meningitis): 33.3%	2015 Cefotaxime NS (non-meningitis): 30.6%				No change		2010 Erythromycin NS: 100%	2015 Erythromycin NS: 92.4%		Decrease		2010 Levofloxacin NS: 0.0%	2015 Levofloxacin NS: 1.5%		No change
			Penicillin NS (meningitis): 96.0%	Penicillin NS (meningitis): 96.7%		No change		Cefotaxime NS (meningitis): 95.8%	Cefotaxime NS (meningitis): 59.7%				Decrease							Vancomycin NS: 0.0%	Vancomycin NS: 0.0%		No change
8	Diawara, 2017 [18]	Morocco	Penicillin NS: 31.0%	Penicillin NS: 13.0%	χ^2^ *P* = 0.009	Decrease																	
9	Echaniz-Aviles, 2019 [19]	Mexico	Penicillin R (non-meningitis): 16.6%	Penicillin R (non-meningitis): 10.0%		Decrease		Cefotaxime R (non-meningitis): 2.2%	Cefotaxime R (non-meningitis): 14.4%				Increase		Erythromycin R: 4.2%	Erythromycin R: 30.2%		Increase		Chloramphenicol R: 1.0%	Chloramphenicol R: 20.8%		Increase
			Penicillin R (meningitis): 0.0%	Penicillin R (meningitis): 50.0%		Increase		Cefotaxime R (meningitis): 0.0%	Cefotaxime R (meningitis): 0.0%				No change							Trimethoprim/sulfamethoxazole R: 11.5%	Trimethoprim/sulfamethoxazole R: 45.8%		Increase
11	Gagetti, 2017 [21]	Argentina	1993 Penicillin R (19A): 0%	2014 Penicillin R (19A): 87.5%		Increase																	
12	Gaviria-Agudelo, 2017 [41]	USA	Penicillin R by meningitis breaking point: 53.9%	Penicillin R by meningitis breaking point: 35.3%	*P* < 0.015	Decrease		Cefotaxime R by meningitis breaking point: 9.3%	Cefotaxime R by meningitis breaking point: 1.7%		Not significant		No change										
			Penicillin R by isolate breaking point: 0%	Penicillin R by isolate breaking point: 8.2%	*P* < 0.008	Increase		Cefotaxime R by isolate breaking point: 0.7%	Cefotaxime R by isolate breaking point: 0%		Not significant		No change										
13	Ho, 2019 [22]	Hong Kong, China	Penicillin NS (non-meningitis): 2.3%	Penicillin NS (non-meningitis): 3.4%	χ^2^ *P* < 0.001	Increase									Erythromycin NS: 84.9%	Erythromycin NS: 69.9%	χ^2^ *P* = 0.226	No change					
			Penicillin NS (meningitis): 46.5%	Penicillin NS (meningitis): 13.8%		Decrease																	
15	Huang, 2019 [24]	Taiwan, China	Penicillin S by MIC (ug/mL): <0.06 = 91.2%, 0.1-1.0 = 42.7% 2 = 72.1% 4 = 94.5% 8 = 99.4%	Penicillin S by MIC (ug/mL): <0.06 = 100.0% 0.12-1.0 = 51.3% 2 = 66.7% 4 = 82.1% 8 = 100.0%	χ^2^ *P* < 0.001	No change		Ceftriaxone NS: 1.2%	Ceftriaxone NS: 10.3%		χ^2^ *P* < 0.001		Increase		Erythromycin NS: 97.9%	Erythromycin NS: 87.2%	χ^2^ *P* < 0.001	Decrease		Moxifloxacin NS: 1.2%	Moxifloxacin NS: 12.8%	χ^2^ *P* < 0.001	Increase
																				Trimethoprim/Sulfamethoxazole NS: 81.5%	Trimethoprim/Sulfamethoxazole NS: 56.4%		Decrease
																				Vancomycin NS: 0.0%	Vancomycin NS: 0.0%		No change
21	Mott, 2019 [29]	Brazil	Penicillin (non-meningitis) R: 0.0% (n = 0)	Penicillin (non-meningitis) R: 0.0% (n = 0)		No change		Ceftriaxone (non-meningitis) R: 0.0% (n = 0)	Ceftriaxone (non-meningitis) R: 0.0% (n = 0)				No change		Erythromycin R: 0.0% (n = 0)	Erythromycin R: 71.0% (n = 22)		Increase		Meropenem R: 0.0% (n = 0)	Meropenem R: 16.7% (n = 5)		Increase
			Penicillin (meningitis) R: 80.0% (n = 4)	Penicillin (meningitis) R: 76.7% (n = 23)		No change		Ceftriaxone (meningitis) R: 0.0% (n = 0)	Ceftriaxone (meningitis) R: 40.0% (n = 12)				Increase							Tetracycline R: 0.0% (n = 0)	Tetracycline R: 29.0% (n = 9)		Increase
																				Trimethoprim-Sulfamethoxazole R: 20.0% (n = 1)	Trimethoprim-Sulfamethoxazole R: 25.8% (n = 8)		No change
22	Neves, 2018 [40]	Brazil	Penicillin NS: 24.0% (n = 30)	Penicillin NS: 39.0% (n = 51)	*P* = 0.01	Increase																	
1	Ando, 2020 [11]	Japan	2010 Penicillin NS: 6.3%	2017 Penicillin NS: 1.6%		Decrease		2010 Cefotaxime NS: 9.7%	2017 Cefotaxime: 1.4%				Decrease		2010 Erythromycin: 94.1%	2017 Erythromycin: 83.6%		Decrease		2010 Meropenem: 31.4%	2017 Meropenem: 18.9%		Decrease
								Ceftriaxone NS: 2.8%	Ceftriaxone:1.6.0%				No change							Vancomycin: 0.0%	Vancomycin: 0.0%		No change
								Cefepime NS: 29.2%	Cefepime: 3.9%				Decrease							Levofloxacin: 1.7%	Levofloxacin: 1.2%		No change
2	Ben-Shimol, 2018 [12]	Israel	Penicillin R: 40.5% ± 8.0%	Penicillin R: 9.6% ± 7.4%	Rate / risk difference = 30.9%	Decrease		Cefotaxime R: 5.0% ± 0.8%	Cefotaxime R: 0.0%		Rate / risk difference = 5.0%		Decrease										
4	Berger, 2019 [14]	Israel	Blood isolates Penicillin NS: 19%	Blood isolates Penicillin NS: 7%	*P* = 0.009	Decrease		Ceftriaxone IR: 4%	Ceftriaxone IR: 2%		*P* < 0.47		No change										
			Penicillin R: 3%	Penicillin R:2%	*P* = non significant	No change																	
			CSF isolates: Penicillin NS: 1%	CSF isolates: Penicillin NS: 0%	No *P*-value calculated	No change																	
10	Furuya, 2017 [20]	Japan	Benzylpenicillin NS ratio: 0.8	Benzylpenicillin NS ratio: 4.9		No change		Cefdinir NS ratio: 53.0	Cefdinir NS ratio: 44.3				Decrease		Clarithromycin NS ratio: 85.6	Clarithromycin NS ratio: 95.1		Increase		Imipenem NS ratio: 10.2	Imipenem NS ratio: 27.9		Increase
			Amoxicillin NS ratio: 1.4	Amoxicillin NS ratio: 1.6		No change														Meropenem NS ratio: 12.6	Meropenem NS ratio: 24.6		Increase
			Clavulanic acid-amoxicillin NS ratio: 0.9	Clavulanic acid-amoxicillin NS ratio: 1.6		No change														Levofloxacin NS ratio: 0.5	Levofloxacin NS ratio: 0.0		No change
17	Koutouzis, 2018 [26]	Greece	Penicillin G R: 8.5% (n = 4)	Penicillin G R: 50.7% (n = 38)	χ^2^ *P* = 0.001	Increase		Cefotaxime R: 2.1% (n = 1)	Cefotaxime R: 30.7% (n = 23)		χ^2^ *P* < 0.001		Increase		Erythromycin R: 6.4% (n = 3)	Erythromycin R: 80.0% (n = 60)	χ^2^ *P* < 0.001	Increase		Clindamycin R: 6.4% (n = 3)	Clindamycin R: 65.3% (n = 49)	χ^2^ *P* < 0.001	Increase
																				Tetracycline R: 19.1% (n = 9)	Tetracycline R: 60.0% (n = 45)	χ^2^ *P* < 0.001	Increase
																				Chloramphenicol R: 4.3% (n = 2)	Chloramphenicol R: 0.0% (n = 0)	χ^2^ *P* < 0.001	Decrease
24	Quirk, 2018 [30]	Iceland	Penicillin NS: 36.9% (n = 116)	Penicillin NS: 30.4% (n = 70)	*P* = 0.121	No change									Erythromycin NS: 37.6% (n = 118)	Erythromycin NS: 33.5% (n = 77)	*P* = 0.366	No change		Chloramphenicol R: 1.6% (n = 5)	Chloramphenicol R: 2.2% (n = 5)	*P* = 0.750	No change
																				Tetracycline R: 34.7% (n = 109)	Tetracycline R: 27.0% (n = 62)	*P* = 0.062	No change
																				Clindamycin R: 32.5% (n = 102)	Clindamycin R: 24.3% (n = 56)	*P* = 0.045	Decrease
																				Trimethoprim-sulfamethoxazole R: 42.7% (n = 134)	Trimethoprim-sulfamethoxazole R: 21.3% (n = 49)	*P* < 0.001	Decrease
25	Ricketson, 2018 [31]	Canada	Penicillin (non-meningitis) R: 0.0%	Penicillin (non-meningitis) R: 2.9%		Increase														Nonsusceptibility to at least one antibiotic used for parenteral treatment of S. pneumoniae infections (including beta-lactams, quinolones and vancomycin): 9.6%	Nonsusceptibility to at least one antibiotic used for parenteral treatment of S. pneumoniae infections (including beta-lactams, quinolones and vancomycin): 17.7%	*P* = 0.292	Increase
23	Quirk, 2018 [30]	Iceland	Penicillin NS: 15.0% (n = 149)	Penicillin NS: 16.7% (n = 338)	*P* = 0.268	No change									Erythromycin R: 17.6%	Erythromycin R: 13.7%	*P* = 0.007	Decrease		Multidrug resistant R: 15.2% (n = 151)	Multidrug resistant R: 12.4% (n = 251)	*P* = 0.030	No change
28	Siira, 2020 [34]	Norway	Penicillin NS: 1.5% (n = 27)	Penicillin NS: 5.3% (n = 151)		No change		Ceftriaxone R: 0.0% (n = 0)	Ceftriaxone R: 0.0% (n = 0)				No change		Erythromycin R: 10.0% (n = 178)	Erythromycin R: 4.7% (n = 135)		Decrease		Multidrug resistant R: 0.7% (n = 12)	Multidrug resistant R: 3.1% (n = 88)		Increase
								Cefotaxime R: 0.0% (n = 0)	Cefotaxime R: 0.0% (n = 0)				No change							Trimethoprim/sulfamethoxazole R: 0.0% (n = 0)	Trimethoprim/sulfamethoxazole R: 6.2% (n = 177)		Increase
																				Clindamycin R: 1.1% (n = 20)	Clindamycin R: 4.3% (n = 97)		Increase
																				Tetracycline R: 1.7% (n = 31)	Tetracycline R: 4.7% (n = 132)		Increase
18	Lo, 2019 [27]	Hong Kong, Israel, Malawi, South Africa, The Gambia, and the USA	All IPD isolates	All IPD isolates	All IPD isolates, adjusted linear regression										All IPD isolates	All IPD isolates	All IPD isolates, adjusted linear regression			All IPD isolates	All IPD isolates	All IPD isolates, adjusted linear regression	
			Penicillin NS: 49.1% (n = 774)	Penicillin NS: 34.0% (n = 277)	*P* < 0.0001	Decrease									Erythromycin NS: 23.9% (n = 377)	Erythromycin NS: 15.0% (n = 122)	*P* < 0.0001	Decrease		Chloramphenicol NS: 4.9% (n = 78)	Chloramphenicol NS: 4.8% (n = 39)	*P* = 0.93	No change
																				Cotrimoxazole NS: 70.1% (n = 1118)	Cotrimoxazole NS: 49.0% (n = 399)	*P* < 0.0001	Decrease
																				Tetracycline NS:28.3% (n = 446)	Tetracycline NS:18.2% (n = 148)	*P* < 0.0001	Decrease
																				Multidrug resistance NS: 26% (n = 410)	Multidrug resistance NS: 15% (n = 125)	*P* < 0.0001	Decrease
			Non-VT IPD isolates only	Non-VT IPD isolates only	Non-VT IPD isolates only, adjusted linear regression										Non-VT IPD isolates only	Non-VT IPD isolates only	Non-VT IPD isolates only, adjusted linear regression			Non-VT IPD isolates only	Non-VT IPD isolates only	Non-VT IPD isolates only, adjusted linear regression	
			Penicillin NS: 20.8% (n = 52)	Penicillin NS: 29.4% (n = 169)	*P* = 0.0016	Increase									Erythromycin NS: 1.2% (n = 3)	Erythromycin NS: 11.3% (n = 65)	*P* = 0.031	Increase		Chloramphenicol NS: 5.6% (n = 14)	Chloramphenicol NS: 5.4% (n = 31)	*P* = 0.93	No change
																				Cotrimoxazole NS: 48.2% (n = 120)	Cotrimoxazole NS: 39.0% (n = 224)	*P* = 0.021	Decrease
																				Tetracycline NS:14.4% (n = 36)	Tetracycline NS:13.9% (n = 80)	*P* = 0.83	No change
																				Multidrug resistance NS: 8.4% (n = 21)	Multidrug resistance NS: 10.3% (n = 59)	*P* = 0.79	No change

PCV – pneumococcal conjugate vaccine, IPD – invasive pneumococcal disease, VT – vaccine type, NS – non-susceptible, R – resistant, y – year

There were 19 results from 22 studies on the changes in cephalosporin resistance or non-susceptibility. Among the 19 results, six (32%) studies observed declines in AMR rates in the post-PCV10/13 period, four (21%) observed increases and nine (47%) observed no change (**Table 1**).

There were 12 results from 13 studies on the changes in macrolide resistance or non-susceptibility. Among the 12 results, four (33%) studies observed declines in AMR rates in the post-PCV10/13 period, six (50%) observed increases and two (17%) observed no change (**Table 1**).

There were 39 results from 14 studies on the changes in other antibiotic resistance or non-susceptibility, which included multidrug resistance. Among the 39 results, nine (23%) studies observed declines in AMR rates in the post-PCV10/13 period, 16 (41%) observed increases and 14 (36%) observed no change (**Table 1**).

### Changes in AMR rates for IPD isolates by population factors

Changes in AMR rates for IPD isolates by DDD, country World Bank income status, PCV valency and time since vaccine introduction are shown in **Table 2**. There were no obvious differences in the number of studies reporting an increase vs a decrease in AMR rates for any of the population factors which were assessed.

**Table 2:** Percentage of estimates by the direction of change (increase, decrease or no change) in antimicrobial resistance rates for IPD isolates by the population level risk factor and family of antibiotic

	Penicillins				Macrolides				Cephalosporins				Others			
Increase, n (%*)	Decrease, n (%*)	***P*-value†**	**No change, n (%**‡**)**	Increase, n (%*)	Decrease, n (%*)	***P*-value†**	**No change, n (%**‡**)**	Increase, n (%*)	Decrease, n (%*)	***P*-value†**	**No change, n (%**‡**)**	Increase, n (%*)	Decrease, n (%*)	***P*-value†**	**No change, n (%)**
**Defined daily doses per 1000 inhabitants per day for any antibiotic§**																
High, 20+	1 (50)	1 (50)	0.81	5 (71)	0	3 (100)	0.08	3 (50)	1 (33)	2 (67)	0.08	1 (25)	7 (70)	3 (30)	0.45	5 (33)
Low, <20	3 (60)	2 (40)		2 (28)	2 (67)	1 (33)		1 (25)	3 (100)	0		0	6 (86)	1 (14)		1 (13)
**World Bank Country income status (2021 classification)**																
LIC	0	0	0.89	2 (100)	0	0	0.20	0	0	0	0.47	0	0	0	0.34	1 (100)
LMIC	0	0		0	0	0		0	0	0		0	0	0		0
UMIC	7 (54)	6 (46)		3 (19)	3 (60)	2 (40)		5 (50)	3 (75)	1 (25)		1 (20)	7 (88)	1 (13)		4 (33)
HIC	2 (50)	2 (50)		5 (56)	1 (20)	4 (80)		4 (44)	2 (50)	2 (50)		1 (20)	9 (69)	4 (31)		5 (28)
**PCV valency**																
PCV10	3 (50)	3 (50)	0.86	2 (25)	1 (50)	1 (50)	0.75	3 (60)	2 (100)	0	0.21	0	4 (100)	0	0.214	1 (20)
PCV13	6 (54)	5 (45)		8 (42)	3 (38)	5 (62)		6 (43)	3 (50)	3 (50)		2 (25)	12 (71)	5 (29)		9 (35)
**Time since PCV introduction**																
<3 y	3 (60)	2 (40)	0.71	4 (44)	1 (25)	3 (75)	0.43	2 (33)	2 (100)	0	0.21	0	4 (100)	0	0.10	3 (43)
3+ y	6 (50)	6 (50)		6 (33)	3 (50)	3 (50)		7 (54)	3 (50)	3 (50)		2 (25)	12 (57)	9 (43)		11 (34)

PCV – pneumococcal conjugate vaccine, IPD – invasive pneumococcal disease, y – year

*Denominator includes only estimates which increased or decreased in the post-PCV period and excludes estimates which did not change.

†*P*-value for χ^2^ test comparing increased vs. decreased.

‡Denominator includes all estimates; increased, decreased and no change, in the post-PCV period.

§Data on the defined daily doses of antibiotics consumed per 1000 inhabitants per day for any antibiotic for countries were extracted from WHO report.

### Changes in AMR rates for OM isolates

There was only one study documenting changes in AMR rates and this was for penicillin resistance or non-susceptibility for which there was no observed change (**Table 3**). The numbers of studies assessing changes in AMR rates for OM isolates were too few for sub-group analysis by population factors.

**Table 3:** Results of studies displaying the rates of resistant or non-susceptible otitis media isolates in the pre- and post-PCV10/13 periods

ID	Author	Country	Penicillin family (penicillin, amoxicillin, amoxicillin-clavulanate (augmentin), ampicillin, dicloxacillin, nafcillin, and piperacillin-tazobactam (zosyn))				Cephalosporin family (all antibiotics starting with Cef)				Macrolide family (erythromycin, roxithromycin, azithromycin and clarithromycin)				Other				
**Pre-PCV antibiotic resistance**	**Post-PCV antibiotic resistance**	***P*-value for comparison of pre- vs post- antibiotic resistnace**	**Increase, decrease either *P* < 0.05 or +/− 5%**	**Pre-PCV antibiotic resistance**	**Post-PCV antibiotic resistance**	**Percent change in antibiotic resistance**	**Increase, decrease either *P* < 0.05 or +/− 5%**	**Pre-PCV antibiotic resistance**	**Post-PCV antibiotic resistance**	**Percent change in antibiotic resistance**	**Increase, decrease either *P* < 0.05 or +/− 5%**	**Pre-PCV antibiotic resistance**	**Post-PCV antibiotic resistance**	**Percent change in antibiotic resistance**	**Increase, decrease either *P* < 0.05 or +/− 5%**
14	Hoshino, 2017 [23]	Japan	Penicillin R: 25%	Penicillin R: 4.5%	*P* = 0.038	No change												

PCV – pneumococcal conjugate vaccine, R – resistant

### Changes in AMR rates for NPC isolates

There were four results from two studies on the changes in penicillin resistance or non-susceptibility. Among the four results, one study observed a decline in AMR rates in the post-PCV10/13 period, none observed an increase and three (75%) observed no change (**Table 4**).

**Table 4:** Results of studies displaying the rates of resistant or non-susceptible nasopharyngeal carriage isolates in the pre- and post-PCV10/13 periods

ID	Author	Country	Penicillin family (penicillin, amoxicillin, amoxicillin-clavulanate (augmentin), ampicillin, dicloxacillin, nafcillin, and piperacillin-tazobactam (zosyn))					Cephalosporin family (all antibiotics starting with Cef)									Macrolide family (erythromycin, roxithromycin, azithromycin and clarithromycin)					Other						
**Pre-PCV antibiotic resistance**	**Post-PCV antibiotic resistance**	***P* value for comparison of pre- vs post- antibiotic resistance**	**Increase, decrease either *P* < 0.05 or +/− 5%**		**Pre-PCV antibiotic resistance**			**Post-PCV antibiotic resistance**		**Percent change in antibiotic resistance**		**Increase, decrease either *P* < 0.05 or +/− 5%**		**Pre-PCV antibiotic resistance**		**Post-PCV antibiotic resistance**	**Percent change in antibiotic resistance**	**Increase, decrease either *P* < 0.05 or +/− 5%**	**Pre-PCV antibiotic resistance**	**Post-PCV antibiotic resistance**		**Percent change in antibiotic resistance**		**Increase, decrease either *P* < 0.05 or +/− 5%**
16	Kobayashi, 2020 [25]	Kenya	Pneumococcal isolates from Kibera children	Pneumococcal isolates from Kibera children	Pneumococcal isolates from Kibera children				Pneumococcal isolates from Kibera children	Pneumococcal isolates from Kibera children		Pneumococcal isolates from Kibera children				Pneumococcal isolates from Kibera children			Pneumococcal isolates from Kibera children	Pneumococcal isolates from Kibera children		Pneumococcal isolates from Kibera children	Pneumococcal isolates from Kibera children		Pneumococcal isolates from Kibera children		
			Penicillin R: 2.4% (n = 12)	Penicillin R: 2.7% (n = 12)	χ^2^ *P* = 0.618	No change			Ceftriaxone R: 0% (n = 0)	Ceftriaxone R: 0% (n = 0)		χ^2^ *P* = NA		No change		Erythromycin R: 1.6% (n = 8)			Erythromycin R: 3.8% (n = 17)	χ^2^ *P* = 0.042	No change	Chloramphenicol R: 1.8% (n = 9)	Chloramphenicol R: 3.2% (n = 14)		χ^2^ *P* = 0.182	No change	
																						Levofloxacin R: 0% (n = 0)	Levofloxacin R: 0% (n = 0)		χ^2^ *P* = NA	No change	
																						Tetracycline R: 17.0% (n = 85)	Tetracycline R: 12.8% (n = 57)		χ^2^ *P* = 0.187	No change	
																						Cotrimoxazole R: 86.2% (n = 430)	Cotrimoxazole R: 90.1% (n = 401)		χ^2^ *P* = 0.029	Increase	
																						Clindamycin R: 0.2% (n = 1)	Clindamycin R: 2.7% (n = 12)		χ^2^ *P* = 0.001	Increase	
			Pneumococcal isolates from Lwak children	Pneumococcal isolates from Lwak children	Pneumococcal isolates from Lwak children		Pneumococcal isolates from Lwak children				Pneumococcal isolates from Lwak children	Pneumococcal isolates from Lwak children				Pneumococcal isolates from Lwak children		Pneumococcal isolates from Lwak children		Pneumococcal isolates from Lwak children		Pneumococcal isolates from Lwak children	Pneumococcal isolates from Lwak children	Pneumococcal isolates from Lwak children			
			Penicillin R: 1.8% (n = 3)	Penicillin R: 0.0% (n = 0)	χ^2^ *P* = 0.235	No change	Ceftriaxone R: 0.0% (n = 0)				Ceftriaxone R: 0.0% (n = 0)	χ^2^ *P* = NA		No change		Erythromycin R: 0.6% (n = 1)		Erythromycin R: 0.0% (n = 0)		χ^2^ *P* = 0.474	No change	Chloramphenicol R: 2.6% (n = 4)	Chloramphenicol R:3.3% (n = 6)	χ^2^ *P* = 0.757		No change	
																						Levofloxacin R: 0.0% (n = 0)	Levofloxacin R: 0.0% (n = 0)	χ^2^ *P* = NA		No change	
																						Tetracycline R: 18.4% (n = 30)	Tetracycline R: 11.6% (n = 21)	χ^2^ *P* = 0.001		Decrease	
																						Cotrimoxazole R: 5.6% (n = 9)	Cotrimoxazole R: 5.5% (n = 10)	χ^2^ *P* = 1.00		No change	
																						Clindamycin R: 0.0% (n = 0)	Clindamycin R: 0.0% (n = 0)	χ^2^ *P* = NA		No change	
31	Turner, 2020 [37]	Cambodia	Penicillin R: 81.0% (n = 265)	Penicillin R: 65.6% (n = 231)		Decrease		Ceftriaxone R: 18.3% (n = 60)			Ceftriaxone R: 11.1% (n = 39)				Decrease		Erythromycin R: 52.3% (n = 171)		Erythromycin R: 47.2% (n = 166)		Decrease	Multi-drug resistant R: 63.9% (n = 241)	Multi-drug resistant R: 63.9% (n = 225)		`		No change
																						Chloramphenicol R: 12.2% (n = 40)	Chloramphenicol R: 11.1% (n = 39)				No change
																						Clindamycin R: 40.4% (n = 132)	Clindamycin R: 33.5% (n = 118)				Decrease
																						Co-trimoxazole R: 73.4% (n = 240)	Co-trimoxazole R: 71.0% (n = 250)				No change
																						Tetracycline R: 87.8% (n = 287)	Tetracycline R: 81.0% (n = 285)				No change
19	Mayanskiy, 2017 [38]	Russia															2010-2011		2016	*P* = 0.001		2010-2011	2016				
																	Erythromycin R: 24.5% (n = 46)		Erythromycin R: 42.3% (n = 60)		Increase	Oxacillin R: 21.3% (n = 40)	Oxacillin R: 35.9% (n = 106)		*P* = 0.001		Increase
																						Clindamycin R: 19.9% (n = 46)	Clindamycin R: 23.4% (n = 69)		*P* = 0.482		No change
																						Sulfamethoxazole/ Trimethoprim R: 59.1% (n = 110)	Sulfamethoxazole/ Trimethoprim R: 37.6% (n = 111)		*P* = 0.001		Decrease
																						Chloramphenicol R: 14.6% (n = 6)	Chloramphenicol R: 4.7% (n = 14)		*P* = 0.002		Decrease
																						Tetracycline R: 60.0% (n = 15)	Tetracycline R: 29.0% (n = 85)		*P* = 0.001		Decrease
20	Mayanskiy, 2019 [28]	Russia															2010-2011		2017	*P* = 0.004	Increase	2010-2011	2017				
																	Erythromycin R: 27.0% (n = 24)		Erythromycin R: 35.8% (n = 38)			Oxacillin R: 20.0% (n = 18)	Oxacillin R: 31.1% (n = 33)		*P* = 0.009		Increase
																						Clindamycin R: 25.0% (n = 56)	Clindamycin R: 28.3% (n = 30)		*P* = 0.004		Increase
																						Sulfamethoxazole/ Trimethoprim R: 64.0% (n = 56)	Sulfamethoxazole/ Trimethoprim R: 41.5% (n = 44)		*P* = 0.006		Increase
																						Chloramphenicol R: Not tested	Chloramphenicol R: 0.;9% (n = 1)		*P* = NA		
																						Tetracycline R: Not tested	Tetracycline R: 32.1% (n = 34)		*P* = NA		
																						Multi-drug resistant R: 26.0% (n = 23)	Multi-drug resistant R: 27.4% (n = 29)		*P* = 0.225		No change

PCV – pneumococcal conjugate vaccine, IPD – invasive pneumococcal disease, NA – not available, R – resistant

There were three results from two studies on the changes in cephalosporin resistance or non-susceptibility. Among the three results, one study observed a decline in AMR rates in the post-PCV10/13 period, none observed an increase and two (67%) observed no change (**Table 4**).

There were six results from four studies on the changes in macrolide resistance or non-susceptibility. Among the six results, two (33%) studies observed declines in AMR rates in the post-PCV10/13 period, two (33%) observed increases and two (33%) observed no change (**Table 4**).

There were 25 results from four studies on the changes in other antibiotic resistance or non-susceptibility, which included multidrug resistance. Among the twenty results, five (20%) studies observed declines in AMR rates in the post-PCV10/13 period, six (24%) observed increases, and 14 (56%) observed no change (**Table 4**).

The numbers of results were too small to assess changes in AMR rates for NPC isolates by population factors.

### Changes in AMR rates for other (sputum or mixed invasive and non-invasive pneumococcal) isolates

There were five results from two studies on the changes in penicillin resistance or non-susceptibility. Among the five results, one study observed a decline in in AMR rates in the post-PCV10/13 period, one observed an increase, and three (60%) observed no change (**Table 5**).

**Table 5:** Results of studies displaying the rates of resistant or non-susceptible other (sputum or mixed invasive and non-invasive pneumococcal) isolates in the pre- and post-PCV10/13 periods

ID	Author	Country	Penicillin family (penicillin, amoxicillin, amoxicillin-clavulanate (augmentin), ampicillin, dicloxacillin, nafcillin, and piperacillin-tazobactam (zosyn))				Cephalosporin family (all antibiotics starting with Cef)				Macrolide family (erythromycin, roxithromycin, azithromycin and clarithromycin)				Other			
**Pre-PCV antibiotic resistance**	**Post-PCV antibiotic resistance**	***P* value for comparison of pre- vs post- antibiotic resistance**	**Increase, decrease either *P* < 0.05 or +/− 5%**	**Pre-PCV antibiotic resistance**	**Post-PCV antibiotic resistance**	**Percent change in antibiotic resistance**	**Increase, decrease either *P* < 0.05 or +/− 5%**	**Pre-PCV antibiotic resistance**	**Post-PCV antibiotic resistance**	**Percent change in antibiotic resistance**	**Increase, decrease either *P* < 0.05 or +/− 5%**	**Pre-PCV antibiotic resistance**	**Post-PCV antibiotic resistance**	**Percent change in antibiotic resistance**	**Increase, decrease either *P* < 0.05 or +/− 5%**
26	Shoji, 2017 [32]	Japan	Penicillin R: 10.3% (n = 62)	Penicillin R: 5.1% (n = 25)	*P* = 0.521	No change												
27	Sivhonen, 2017 [33]	Finland													Isolates among children <5 y	Isolates among children <5 y		
															Multidrug resistance R: 9.4% (n = 3)	Multidrug resistance R: 25% (n = 2)	Not statistically significant	No change
															Isolates among people ≥5 y	Isolates among people ≥5 y		
															Multidrug resistance R: 4.3% (n = 8)	Multidrug resistance R: 1.6% (n = 3)	Not statistically significant	No change
29	Suzuki, 2017 [35]	Japan					Cefotaxime NS: 7.2%	Cefotaxime NS: 0.7%	*P* = 0.007	Decrease	Clarithromycin NS: 94.0%	Clarithromycin NS: 84.5%	*P* = 0.177	No change	Benzylpenicillin NS: 88.0%	Benzylpenicillin NS: 53.2%	*P* < 0.001	Decrease
											Azithromycin NS: 95.2%	Azithromycin NS: 89.9%	*P* = 0.165	No change	Imipenem NS: 22.7%	Imipenem NS: 17.3%	*P* = 0.605	No change
															Levofloxacin NS: 0.0%	Levofloxacin NS: 0.0%	NA	No change
30	Toda, 2018 [36]	Japan	Penicillin (oral administration, non-meningitis syndrome) among adults NS: 54.5% (n = 198)	Penicillin (oral administration, non-meningitis syndrome) among adults NS: 56.5% (n = 257)		No change	Ceftriaxone (non-meningitis) among adults NS: 1.9% (n = 7)	Ceftriaxone (non-meningitis) among adults NS: 6.6% (n = 30)		No change					Imipenem among adults NS: 18.5% (n = 67)	Imipenem among adults NS: 14.3% (n = 65)		Decrease
			Penicillin (oral administration, non-meningitis syndrome) among children NS: 70.8% (n = 167)	Penicillin (oral administration, non-meningitis syndrome) among children NS: 64.5% (n = 165)		Decrease	Ceftriaxone (non-meningitis) among children NS: 2.1% (n = 5)	Ceftriaxone (non-meningitis) among children NS: 7.4% (n = 19)		Increase					Imipenem among children NS: 18.6% (n = 44)	Imipenem among children NS: 15.2% (n = 39)		Decrease
			Penicillin (parenteral administration, non-meningitis syndrome) among adults NS: 1.1% (n = 4)	Penicillin (parenteral administration, non-meningitis syndrome) among adults NS: 5.9% (n = 29)		No change	Cefotaxime (non-meningitis) among adults NS: 2.8% (n = 10)	Cefotaxime (non-meningitis) among adults NS: 4.2% (n = 19)		No change					Meropenem among adults NS: 14.9% (n = 54)	Meropenem among adults NS: 14.9% (n = 68)		No change
			Penicillin (parenteral administration, non-meningitis syndrome) among children NS: 0.4% (n = 1)	Penicillin (parenteral administration, non-meningitis syndrome) among children NS: 6.3% (n = 16)		Increase	Cefotaxime (non-meningitis) among children NS: 1.7% (n = 4)	Cefotaxime (non-meningitis) among children NS: 4.7% (n = 12)		No change					Meropenem among children NS: 14.4% (n = 34)	Meropenem among children NS: 16.0% (n = 41)		No change

PCV – pneumococcal conjugate vaccine, NS – non-susceptible, R – resistant

There were five results from two studies on the changes in cephalosporin resistance or non-susceptibility. Among the five results, one study observed a reduction in AMR rates in the post-PCV10/13 period, one observed an increase and three (60%) observed no change (**Table 5**).

There were two results from one study on the changes in macrolide resistance or non-susceptibility, both of which observed no change (**Table 5**).

There were nine results from three studies on the changes in other antibiotic resistance or non-susceptibility, which included multidrug resistance. Among the nine results, three (33%) studies observed reductions in AMR rates in the post-PCV10/13 period and six (67%) observed no change (**Table 5**).

The numbers of results were too small to assess changes in AMR rates for other isolates by population factors.

## DISCUSSION

This systematic review on the impact of PCV10 or PCV13 on AMR in IPD, OM, NPC and other (sputum or mixed invasive and non-invasive pneumococcal) found no evidence of a consistent pattern in change in AMR following introduction of PCV. While most studies found reductions or no change in AMR rates, in a minority of studies AMR increased in the period following PCV use, which varied across studies from one to seven years post PCV introduction. There were variations in antimicrobial use across studies settings, as assessed by methods described in the study and DDD data, which meant that data were too varied to allow a detailed analysis because of heterogeneity between studies.

Both previous and subsequent systematic reviews published on the rates of AMR in IPD, OM and NPS before and after the introduction of PCV10 or PCV13 found that AMR rates declined or stayed the same [7,8]. It may be that after the introduction of PCV, an initial decline in AMR occurs (a “honeymoon period”) that may be followed by a rise in AMR due to high levels of antimicrobial use and replacement with AMR serotypes, the degree of which may depend on the ease of AMR acquisition to different antimicrobial classes [42]. Following the introduction of PCV7, previously rare serotypes, such as 19A, became more prevalent (“serotype replacement”) [43]. Prior to any PCV introduction, AMR was found mostly among paediatric IPD serotypes (6B, 9V, 14, 19F and 23F), which tend to be carried for longer and are all contained within PCV [44]. Replacement serotypes, due to their rarity prior to vaccine introduction, are less likely to be AMR, but AMR could increase in these serotypes as they become more prevalent, as was the case for 19A following the introduction of PCV7.

The comparative heterogeneity in results of this study, which included more data from LMIC populations than previous systematic reviews, may reflect the dynamic nature of AMR. AMR is likely to fluctuate over time due to factors such as the pre-existing level of AMR prior to vaccine introduction and the degree of antimicrobial use in a population, which in turn varies widely between LMIC settings [10,45], if such fluctuations are temporally associated but not causally associated with PCR introduction this may cause an increase, decrease and nullification of apparent AMR rate change. Another possible explanation is bias due to variability in the quality of laboratory AMR assessment. Poor or under-resourced laboratory methods may in theory act to underestimate the prevalence of AMR [46].

A limitation of our literature review is that we were unable to identify individual serotypes in each study and determine AMR rates by serotype, as this information was not reported in the majority of studies. However, a 2016 study from the USA found that post PCV13 introduction among common IPD serotypes 15B/15C (included together since they interconvert), 33F, 22F, and 35B, nearly half (49%) of these isolates were erythromycin resistant (with serotype 35B isolates being predominantly penicillin nonsusceptible with an MIC of two μg/ml) [5]. Another study using data from Hong Kong, Israel, Malawi, South Africa, The Gambia, and the USA showed the five most prevalent serotypes in the PCV13 period varied between countries, with only serotypes 5, 12F, 15B/C, 19A, 33F, and 35B/D common to two or more countries, with distinctive lineages and dissimilar antibiotic resistance profiles in different countries [27]. In non-vaccine serotype isolates, increases were detected in the prevalence of resistance to penicillin from 21% (n = 53/249) vs. 29% (n = 169/575) and erythromycin 1% (n = 3/249) vs. 11% (n = 65/575) in the PCV13 period compared with the pre-PCV period [27].

Another limitation of this review is the heterogeneity between study settings, specifically regarding antimicrobial use and admission and sample collection criteria, therefore a meta-analysis could not be undertaken. In addition, the lack of studies which calculated a percent decline in AMR from the pre-PCV period to the post-PCV10/13 period made it challenging to assess the magnitude of the change across studies. There was some heterogeneity in study design across studies, such as the method for systematic collection of samples included in the study, which may explain the wide variability in reported AMR rates. Although hospital or population based observational studies are the most common method of evaluating vaccine impact, observational studies are inherently susceptible to bias associated with confounders. Confounders that may have influenced studies in this review include regulation of antibiotic use, prescribing practices, changes to health care access or delivery, changes to laboratory methods over time, the AMR break point used, pre-PCV pneumococcal AMR rates, and differences in pneumococcal serotype (and therefore potential differences in AMR by serotype) distribution across settings.

The high variability in rates of AMR, both over time and in epidemiological setting, indicate the need for local centres of laboratory excellence to provide information on local and regional antimicrobial susceptibilities occurring over time since the introduction of relevant vaccines. Extrapolation of the impact of PCV on AMR trends from one country or setting to another should be undertaken with caution due to the high degree and multiplicity of factors driving AMR, such as antibiotic prescribing practices and regulation of antibiotic use.

Although NP samples are a convenient and readily accessible sample, the role of NP samples in monitoring and guiding AMR for invasive disease is not known [47]. Further research is needed to understand the association between NP AMR and IPD AMR. Therefore, an alternative may be to undertake AMR surveillance from invasive isolates from patients with meningitis and empyema. This challenge can be assisted by supporting countries to join the WHO Invasive Bacterial Vaccine Preventable Diseases Surveillance Global Network.

Maintaining quality laboratory standards in low-income countries is often challenging. All countries should be supported to use standard guidelines such as the WHO laboratory guidelines [48] or the Clinical & Laboratory Standards Institute guidelines [49] in order to detect and determine AMR using standard laboratory methods. Additionally, participating in external quality assurance programs are essential. For all countries, there is a need to support countries to adopt the One Health policy and Global Antimicrobial Resistance Surveillance System according to Global Action Plan on Antimicrobial Resistance, in order to combat AMR globally.

There is an urgent need for studies to evaluate programmes of antimicrobial stewardship in areas with high rates of AMR. The introduction of pneumococcal conjugate and Hib vaccines that greatly reduces the probability of a febrile child having invasive bacterial disease should be seen as an opportunity to promote more judicious use of antimicrobial agents.

## CONCLUSIONS

We found no evidence of a systematic pattern of change in AMR following introduction of PCV. While some studies demonstrated modest reductions in AMR following the introduction of PCV10 or PCV13, there was some heterogeneity in these findings. This heterogeneity may be due to a number of reasons including the presence of other, more powerful, drivers of AMR such as indiscriminate use of antimicrobials, treatment adherence and possible presence of counterfeit drugs. In addition, replacement by non-vaccine serotypes with variable resistance patterns to antimicrobials may occur. Variability of AMR levels suggests that antimicrobial policy needs to be informed by local data, which requires quality data from local surveillance systems integrating strong epidemiologic tracking with high quality laboratory units as declines in AMR may be short-lived, may not occur and are setting specific.

## Additional material


Online Supplementary Document
