# Supplementary material for: The impact of the introduction of ten- or thirteen-valent pneumococcal conjugate vaccines on antimicrobial-resistant pneumococcal disease and carriage: A systematic literature review
Source: J Glob Health. 2023 Feb 17;13:05001. doi: 10.7189/jogh.13.05001 (PMC9936452; doi:10.7189/jogh.13.05001)
Supplement: Online Supplementary Document [file jogh-13-05001-s001.pdf]

ONLINE SUPPLEMENTARY DOCUMENT

Title: The impact of the introduction of ten- or thirteen-valent pneumococcal conjugate vaccines on antimicrobial-resistant pneumococcal disease and carriage: a systematic literature review

Authors: Rita Reyburn[1*], Jaclyn Maher[2], Claire von Mollendorf[1,3], Amanda Gwee[1,3,4], Kim Mulholland[1,3,5] and Fiona Russell[1,3]

Table S1. Medline search strategy

MEDLINE Search Strategy
1. exp *Pneumonia/
2. ((lower-respiratory adj3 infection*) or pneumonia or pneumonias or lung-inflammation* or lobitis or nonspecific-inflammatory-lung-disease* or peripneumonia or pleuropneumonia or pleuropneumonitis or pneumonic-lung* or pneumonic-pleurisy or pneumonic-pleuritis or pneumonitides or pneumonitis or pulmonal-inflammation* or pulmonary-inflammation* or pulmonic-inflammation*).tw,kf.
3. *pneumococcal infections/
4. *Streptococcus infections/
5. 1 or 2 or 3 or 4
6. exp *Pneumococcal Vaccines/
7. exp *Immunization/
8. (pnu-im?une or pnuim?une or pcv10 or pcv-10 or pcv13 or pcv-13 or prevenar13 or prevenar-13 or prevnar13 or prevnar-13 or synflorix).tw,kf.
9. ((10-valent or ten-valent or 13-valent or thirteen-valent) and (pneumococcal adj5 vaccine*)).tw,kf.
10. Immunization Programs/
11. 6 or 7 or 8 or 9 or 10
12. *pharynx/ or exp *nasopharynx/
13. exp *Otitis Media/
14. *Carrier State/

15. exp *Microbial Sensitivity Tests/
16. exp *Drug Resistance, Microbial/
17. (resistance or resistant or susceptib* or sensitivit* or nonsusceptib* or non-susceptib* or nasopharyn* or pharyn* or carrier* or carriage* or otitis-media or invasive).tw,kf.
18. 12 or 13 or 14 or 15 or 16 or 17
19. 5 and 11 and 18
20. randomized controlled trial.pt.
21. exp randomized controlled trial/
22. exp case-control studies/
23. (exp animals/ or (rat or rats or mouse or mice or swine or porcine or murine or sheep or lamb or lambs or pig or pigs or piglet or piglets or rabbit or rabbits or cat or cats or dog or dogs or cattle or bovine or monkey or monkeys or trout or marmoset or marmosets).ti.) not human*.sh.
24. limit 19 to (case reports or comment or editorial or guideline or letter or practice guideline)
25. 19 not (20 or 21 or 22 or 23 or 24)
26. . limit 25 to yr="2017 -Current"

Table S2. Study characteristics for studies assessing antimicrobial resistance or non-susceptibility in invasive pneumococcal isolates

Study ID	First author, year of publication	Title	WHO region	Country	Study population	Study design	Data collection period	Age group	Number and type of pneumococcal isolates/infections	Clinical and Laboratory Standards Institute used	PCV10 or PCV13	PCV schedule and catch up	Years in the pre-PCV10/13 period	Years in the post-PCV10/13 period	Quality assessment
**Low income countries**															
7	Darboe, 2019 [17]	Community-acquired Invasive Bacterial Disease in Urban Gambia, 2005-2015: a hospital-based surveillance	AFRO	The Gambia	Rural catchment population of the Medical Research Council Unit, The Gambia at the London School of Hygiene and Tropical Medicine, situated 12 km from the capital, Banjul	Before-after retrospective hospital -based cohort	2005-2015	All ages	242 pneumococcal isolates	Yes	PCV13	3 + 0	2005-2009	2012-2015	Moderate
**Upper middle income countries**															
3	Berezin, 2019 [13]	Invasive pneumococcal disease among hospitalized children in Brazil before and after the	PAHO	Brazil	Urban population <17 years catchment for two hospitals in São Paulo and Uberlândia, each	Before-after retrospective hospital-based cohort	2005-2015	<5years, 6-16 y	260 patients with IPD and positive	Yes	PCV10	2 + 1, no catch up	2005-2009	2011-2015	Moderate

		introduction of a pneumococcal conjugate vaccine			with an average 3000 annual paediatric admissions				pneumococcal isolates						
5	Cassiolato, 2018 [15]	Expansion of the multidrug-resistant clonal complex 320 among invasive Streptcoccus pneumoniae serotype 19A after the introduction of a ten-valent pneumococcal conjugate vaccine in Brazil	PAHO	Brazil	National population of all ages. National Reference laboratory for IPD receiving data from 25 public health laboratories located in 25 Brazilian states	Before-after retrospective laboratory-based cohort	2005-2017	<5 y, 5-49 y, ≥50 y	A total of 9852 IPD isolates, of which 6.8% (n = 673/9852) were serotype 19A	Not stated	PCV10	2 + 1, no catch up	2005-2009	2011-2017	Moderate
6	Cho, 2017 [16]	Redistribution of Streptococcus pneumoniae Serotypes after Nationwide 13-Valent Pneumococcal Conjugate Vaccine Program in Children in Northern Taiwan	WPRO	Taiwan (China)	Urban population <18 y in catchment area od Mackay Memorial Hospital and National Taiwan University Hospital	Before-after retrospective hospital-based cohort	2010-2015	<12 mo, 12-24 mo, 2-5 y, 5-18 y	114 IPD isolates	Yes	PCY13	2 + 1, catch up for 1-5 y	2010-2012 PCV7/PCV13 were provided free-of-charge only for children ≤5 y of age with high risks (i.e. certain underlying diseases or low socioeconomic status) and available privately	2013-2015 PCV13 was publicly funded for all 1-5 y with a catch up campaign	Moderate
8	Diawara, 2017 [18]	Molecular characterization of penicillin non-susceptible Streptococcus pneumoniae isolated before and after pneumococcal conjugate vaccine implementation in Casablanca, Morocco	EMRO	Morocco	Isolates from the Microbiology Laboratory of Ibn Rochd University Hospital Centre of Casablanca, population not described	Before-after retrospective laboratory -based cohort	2007-2014	0-14 y	361 IPD isolates	Yes	PCV13 in 2010, PCV10 in 2013	2 + 1, no catch up	2007-2010	2011-2014	Moderate
9	Echaniz-Aviles, 2019 [19]	Clinical and microbiological characteristics of community-acquired pneumonia associated with Streptococcus pneumoniae in adult patients in Mexico	PAHO	Mexico	Patients who presented to three tertiary care hospitals	Before-after retrospective hospital -based cohort	2000-2015	All ages	96 IPD isolates	Yes	PCV7 in 2008, PCV13 in 2012	Not described	2000-2007	2008-2015	Moderate
11	Gagetti, 2017 [21]	Characterization of Streptococcus pneumoniae invasive serotype 19A isolates from Argentina (1993-2014)	PAHO	Argentina	Samples received by the National Reference Laboratory (NRL) 101 hospitals in 20 provinces and Buenos Aires city	Before-after retrospective laboratory-based cohort	1993-2014	Children <6 y	176 IPD isolates serotype 19A	Yes	PCV13 in 2012	2 + 1, catch-up campaign for 12-24 mo	1993-2011	2012-2014	Moderate
13	Ho, 2019 [22]	Increase in incidence of invasive pneumococcal disease caused by serotype three in children eight years after the introduction of the pneumococcal conjugate vaccine in Hong Kong	WPRO	Hong Kong, China	Isolates captured as clinical laboratories providing service to hospitalised patients	Before-after retrospective laboratory-based cohort	1995-2017	Children <5 y	265 IPD isolates	Yes	PCV7 in 2009, PCV10 in 2010, PCV13 in 2011	3 + 1, catch-up campaign for <2 y	1995-2004 (no PCV available)	2015-2017 (PCV13 only)	Moderate
15	Huang, 2019 [24]	Respiratory pathogens – Some altered antibiotic susceptibility after implementation of pneumococcus vaccine and antibiotic control strategies	WPRO	Taiwan, China	Isolates from children <18 y admitted at Taichung Veterans General Hospital (TCVGH), a tertiary-care referral centre with 1555 beds	Before-after retrospective laboratory -based cohort	2008-2017	Children <18 y	449 IPD isolates	Yes	PCV13 in 2013	2 + 1, catch up <5 y	2008-2012	2016-2017	Moderate
21	Mott, 2019 [29]	Emergence of serotype 19A Streptococcus pneumoniae after PCV10 associated with a ST320 in adult population, in Porto Alegre, Brazil	PAHO	Brazil	Isolates were obtained from three hospitals in Porto Alegre (metropolitan area with more than four million	Before-after retrospective hospital-based cohort	2008-2014	All ages	36 IPD serotype 19A isolates	Yes	PCV10	2 + 1, no catch up	2008-2009	2011-2014	Moderate

					inhabitants), South Brazil										
22	Neves, 2018 [40]	Population structure of Streptococcus pneumoniae colonizing children before and after universal use of pneumococcal conjugate vaccines in Brazil: Emergence and expansion of the MDR serotype 6C-CC386 lineage	PAHO	Brazil	Pneumococcal isolates recovered from samples taken from the nasopharynx of children from seven institutions distributed across five different neighbourhoods in Nitero´i city, Rio de Janeiro state, Brazil	Before-after retrospective health facility-based cohort	2009-2014	All ages	36 IPD serotype 19A isolates	Yes	PCV10	2 + 1, no catch up	2009-2019	2014	Moderate
**High income countries**															
1	Ando, 2020 [11]	The prevalence and antimicrobial susceptibility of Streptococcus pneumoniae isolated from patients at Jikei University Hospitals after the implementation of the pneumococcal vaccination program in Japan	WPRO	Japan	Urban population catchment for four Jikei University Hospitals	Before-after retrospective hospital-based cohort	2009-2017	All ages	5763 IPD isolates	Not stated	PCV13 (following PCV7)	3 + 1, no catch up	3 y (2009-2011)	4 y (2014-2017)	Moderate
2	Ben-Shimol, 2018 [12]	Impact of pneumococcal conjugate vaccines introduction on antibiotic resistance of Streptococcus pneumoniae meningitis in children aged five years or younger, Israel, 2004 to 2016	EURO	Israel	All children <5 y in Israel, 2016 population = 875 000	Before-after prospective, nationwide, population-based and active surveillance	2000-2016	Children <5 y	325 pneumococcal meningitis cases	Yes	PCV13 (following PCV7), 80-95% PCV13 vaccine coverage	2 + 1, no catch up	5 y (2004-2008)	2014-2016	Moderate
4	Berger, 2018 [14]	Paediatric community-acquired bacteræmia, pneumococcal invasive disease and antibiotic resistance fell after the pneumococcal conjugate vaccine was introduced	EURO	Israel	Urban population <18 y from3 children's hospitals in Tel Aviv and Jerusalem	Before-after retrospective hospital-based cohort	2007-2015	<18 y	238 community-acquired bacteræmia cases	Yes	PCV13 (following PCV7), 80-95% PCV13 vaccine coverage	2 + 1,no catch up	2007-2009	2010-2015	Moderate
10	Furuya, 2017 [20]	Impact of the pneumococcal conjugate vaccine on serotype distribution of adult non-invasive Streptococcus pneumoniae isolates in Tokai region, Japan, 2008e2016	WPRO	Japan	Adult patients (>16 y) at 10 hospitals in Gifu or Aichi	Before-after retrospective hospital -based cohort	2008-2016	Adults >16 y	504 IPD	Yes	PCV7 in 2010, PCV13 in 2013	Not described	2008-2009	2015-2016	Moderate
12	Gaviria-Agudelo, 2017 [41]	The Effect of 13-Valent Pneumococcal Conjugate Vaccine on the Serotype Distribution and Antibiotic Resistance Profiles in Children With Invasive Pneumococcal Disease	PAHO	USA	Child patients < 18 y at the Children's Medical Center Dallas (CMCD)	Before-after retrospective laboratory -based cohort	1999-2014	Children <18 y	770 IPD isolates	Yes	PCV7 in 2007, PCV13 in 2010	3 + 1, catch up campaign not described	January 1999-December 2000	January 2011-June 2014	Moderate
17	Koutouzis, 2018 [26]	Characteristics of Streptococcus pneumoniae serotype 19A isolates from children in the pre and post Conjugate Vaccine Era. Single center experience 1986-2015	EURO	Greece	IPD and non-IPD in children from a tertiary care children's hospital located in Athens, Greece	Before-after retrospective hospital-based cohort	1986-2015	States children but does not provide specific age	210 IPD serotype 19A	Yes	PCV7 in 2006, PCV10 and PCV13 in 2010	Not stated	1986-2005	2011-2015	Moderate
24	Quirk, 2018 [30]	Vaccination of Icelandic children with the 10-valent pneumococcal vaccine leads to a significant herd effect among adults in Iceland	EURO	Iceland	All pneumococci isolated from lower respiratory tract samples taken from adults with suspected pneumonia and submitted to the Department of Clinical Microbiology at Landspitali University Hospital in Iceland	Before-after retrospective hospital-based cohort	2009-2017	Adults >18 y	797 IPD isolates	Not stated	PCV13 in 2011	2 + 1	2009-2011	2012-2017	Moderate
25	Ricketson, 2018 [31]	Changes in the nature and severity of invasive pneumococcal disease in children before and after the seven-	PAHO	Canada	All children <18 y of age presenting to a healthcare center in the	Before-after retrospective	2000-2015	Children <18 y	285 IPD isolates	Not stated	PCV13	3 + 1	2000-September 2002	2010-2015	Moderate

		valent and thirteen-valent pneumococcal conjugate vaccine programs in Calgary, Canada			Calgary Zone of Alberta Health Services	hospital-based cohort									
28	Siira, 2020 [34]	Antimicrobial susceptibility and clonality of Streptococcus pneumoniae isolates recovered from invasive disease cases during a period with changes in pneumococcal childhood vaccination, Norway, 2004-2016	EURO	Norway	IPD surveillance data from the Norwegian Surveillance System for Communicable Diseases (MSIS).	Before-after retrospective population-based cohort	2004-2016	All ages	10 239 IPD isolates	Not stated	PCV7 in 2006, PCV13 in 2011	2 + 1	2004-2005	2012-2016	Moderate
**Multiple income status countries in one study**															
18	Lo, 2019 [27]	Pneumococcal lineages associated with serotype replacement and antibiotic resistance in childhood invasive pneumococcal disease in the post-PCV13 era: an international whole-genome sequencing study	WPRO, EURO. AFRO, PAHO	Hong Kong, Israel, Malawi, South Africa, The Gambia, and the USA	IPD in children from hospitals and laboratories in six countries	Multisite before-after retrospective hospital and laboratory -based cohort	1995-2015	Children aged <3 y	2391 IPD isolates	Yes	Varied by country	Varied by country	Varied by country	Varied by country	Moderate

PCV – pneumococcal conjugate vaccine, IPD – invasive pneumococcal disease, AFRO – World Health Organization African Region, AMRO – World Health Organization Region of the Americas, SEARO – World Health Organization South-East Asian Region, EURO – World Health Organization European Region, EMRO – World Health Organization Eastern Mediterranean Region, WPRO – World Health Organization Western Pacific Region, y – years, mo – months

Table S3. Study characteristics for studies assessing antimicrobial resistance or non-susceptibility in otitis media isolates

Study ID	First author, year of publication	Title	WHO Region	Country	Study population	Study design	Data collection period	Age group	Number and type of pneumococcal isolates / infections	Clinical and Laboratory Standards Institute (CLSI) used	PCV10 or PCV13 and vaccine coverage	PCV schedule and catch up	Years in the pre-PCV10/13 period	Years in the post-PCV10/13 period	Quality assessment
**High income countries**															
14	Hoshino, 2017 [23]	Analysis of Streptococcus pneumoniae and Haemophilus influenzae isolated from middle ear fluid before and after the introduction of government subsidies for pneumococcal and H. influenzae type b vaccines in Japan	WPRO	Japan	Isolates from paediatric middle ear fluid samples collected from children aged 15 y or younger at Chiba Children's Hospital	Before-after retrospective laboratory -based cohort	2007-2014	Children <15 y	66 pneumococcal isolates from 820 middle ear fluid samples	Yes	PCV7 in 2010, PCV13 in 2013	Not described	2011-2010	2011-2014	Moderate

PCV – pneumococcal conjugate vaccine, WPRO – World Health Organization Western Pacific Region, y – years

Table S4. Study characteristics for studies assessing antimicrobial resistance or non-susceptibility in nasopharyngeal carriage isolates

Study ID	First author, year of publication	Title	WHO region	Country	Study population	Study design	Data collection period	Age group	Number and type of pneumococcal isolates/ infections	Clinical and Laboratory Standards Institute used	PCV10 or PCV13 and vaccine coverage	PCV schedule & catch up	Years in the pre-PCV10/13 period	Years in the post-PCV10/13 period	Quality assessment
**Lower middle income countries**															
16	Kobayashi, 2020 [25]	Impact of 10-valent pneumococcal conjugate vaccine introduction on pneumococcal carriage and antibiotic susceptibility patterns among children aged <5 years and adults with human immunodeficiency virus infection: Kenya, 2009-2013	AFRO	Kenya	Isolates from cross-sectional pneumococcal carriage surveys were performed in children from Kibera and Lwak, Kenya	Comparison of cross-sectional pneumococcal carriage surveys	2009-2013	Children aged <5 y	Pneumococcal carriage isolates from Kibera children aged <5 y (n = 499 in 2009, n = 445 in 2013). Pneumococcal carriage isolates from Lwak children aged <5 y (n = 163 in 2009, n = 181 in 2013)	Yes	PCV10 in 2011	3 + 0, catch up in selected sites for children <1 y	2009	2013	Moderate
31	Turner, 2020 [37]	Impact of 13-valent pneumococcal conjugate vaccine on colonization and invasive disease in Cambodian children	WPRO	Cambodia	Carriage isolates from children at the outpatient department of the Angkor Hospital for Children is a nongovernmental paediatric hospital	Before-after retrospective hospital-based cohort	2014-2016	Children <5 y	1629 pneumococcal carriage isolates	Yes	PCV13 in 2015	3 + 0, no catch up	2014	2016-2018	Moderate

					located in the north western city of Siem Reap. The hospital, and an associated satellite clinic at Sot Nikom district hospital,										
**Lower middle income countries**															
19	Mayanskiy, 2017 [38]	Serotypes and antimicrobial susceptibility of nasopharyngeal pneumococci isolated from children in 2010-2016: a retrospective cohort study	EURO	Russia	Nasopharyngeal pneumococci isolated from children getting care at the National Medical Research Center of Children's Health (Moscow)	Before-after retrospective hospital -based cohort	2010-2016	Children <18 y	484 carriage isolates	Yes	PCV13 in 2014	2 + 1	2010/11	2016	Moderate
20	Mayanskiy, 2019 [28]	Changing serotype distribution and resistance patterns among paediatric nasopharyngeal pneumococci collected in Moscow, 2010-2017	EURO	Russia	Nasopharyngeal pneumococci isolated from children getting care at the National Medical Research Center of Children's Health (Moscow)	Before-after retrospective hospital -based cohort	2010-2017	Children <5 y	631 carriage isolates	Yes	PCV13 in 2014	2 + 1	2010/11	2017	Moderate
**High income countries**															
23	Quirk, 2019 [30]	Effect of vaccination on pneumococci isolated from the nasopharynx of healthy children and the middle ear of children with otitis media in Iceland	EURO	Iceland	Nasopharyngeal swabs were taken from healthy children attending 15 DCCs, chosen to be representative of the greater Reykjavík area	Before-after health facility-based cross-sectional surveys	2009-2017	Children 1-17 y	3020 carriage isolates	Not stated	PCV13 in 2011	2 + 1	2009-2011	2012-2017	Moderate

PCV – pneumococcal conjugate vaccine, AFRO – World Health Organization African Region, EURO - World Health Organization European Region, WPRO - World Health Organization Western Pacific Region

Table S5. Study characteristics for studies assessing antimicrobial resistance or non-susceptibility in other (sputum or mixed invasive and non-invasive pneumococcal) isolates

Study ID	First author, year of publication	Title	WHO Region	Country	Study population	Study design	Data collection period	Age group	Number and type of pneumococcal isolates/ infections	Clinical and Laboratory Standards Institute used	PCV10 or PCV13 and vaccine coverage	PCV schedule and catch up	Years in the pre-PCV10/13 period	Years in the post-PCV10/13 period	Quality assessment
**High income countries**															
26	Shoji, 2017 [32]	Serotype distribution of Streptococcus pneumoniae isolated from adult respiratory tract infections in nationwide Japanese surveillances 2006-2014	WPRO	Japan	The Japanese Society of Chemotherapy (JSC) nationwide surveillance network of the antimicrobial susceptibility of bacterial respiratory pathogens	Prospective national surveillance	2006-2014	Adults >18 y, median age 73 y	1086 Streptocpccus pneumoniae strains isolated from sputum and specimens of transtracheal aspiration or bronchoscopy	Not stated	PCV13 (following PCV7)	3 + 1, no catch up	2006-2010	2012-2014	Moderate

									in adult respiratory tract infections						
27	Sivhonen, 2017 [33]	Streptococcus pneumoniae antimicrobial resistance decreased in the Helsinki Metropolitan Area after routine 10-valent pneumococcal conjugate vaccination of infants in Finland	EURO	Finland	Clinical invasive (isolated from the blood or the cerebrospinal fluid) and non-invasive (all others, e.g. from ear, eye, nose, throat, maxillary sinus, trachea, bronchus, sputum, or abscess) S. pneumoniae isolates routinely identified at HUSLAB (Hospital District of Helsinki and Uusimaa Laboratory Services)	Before-after retrospective hospital-based cohort	2009-2014	Children <5 y	3040 non-invasive and 1254 IPD isolates	Not stated	PCV10	2 + 1, no catch up campaign described	2009	2014	Moderate
29	Suzuki, 2017 [35]	Impact of the introduction of a 13-valent pneumococcal vaccine on pneumococcal serotypes in non-invasive isolates 2007-2016 at a teaching hospital in Japan	WPRO	Japan	Pneumococcal isolates collected at the Tokyo Medical University Hachioji Medical Center	Before-after retrospective hospital-based cohort	2007-2016	All ages	618 invasive and non-invasive pneumococcal isolates	Yes	PCV7 in 2010, PCV13 in 2013	3 + 1, no catch up	2006-2010	2012-2014	Moderate
30	Toda, 2018 [36]	Laboratory surveillance of antimicrobial resistance and multidrug resistance among Streptococcus pneumoniae isolated in the Kinki region of Japan, 2001e2015: Comparison of the prevalence of drug-resistant strains before and after introduction of conjugated pneumococcal vaccine	WPRO	Japan	Streptococcus pneumoniae collected from 21 medical institutions in the Kinki region that belong to the Study of Bacterial Resistance in the Kinki Region of Japan	Before-after retrospective hospital-based cohort	2001-2016	All ages	4354 pneumococcal isolates	Yes	PCV7 in 2010, PCV13 in 2013	3 + 1, no catch up	2001-2002	2001-2016	Moderate

PCV – pneumococcal conjugate vaccine, IPD – invasive pneumococcal disease, EURO – World Health Organization European Region, WPRO – World Health Organization Western Pacific Region
